# Susceptibility and resistance in the genesis of asbestos-related mesothelioma

**DOI:** 10.4103/0019-5278.43261

**Published:** 2008-08

**Authors:** Claudio Bianchi, Tommaso Bianchi

**Affiliations:** Center for the Study of Environmental Cancer, Italian League Against Cancer, Hospital of Monfalcone, 34074 Monfalcone, Italy

**Keywords:** Asbestos, familial cancer, host factors, immune impairment, mesothelioma, resistance, susceptibility

## Abstract

Asbestos is the principal agent in the etiology of malignant mesothelioma. However, a small proportion of people exposed to asbestos develop mesothelioma. This suggests the role of host factors in the genesis of the tumor. A genetic susceptibility is suggested by the occurrence of more mesothelioma cases among blood-related members of a single family. Such an occurrence reached about 4% in a large mesothelioma series. In some studies, mesothelioma patients showed higher prevalences of additional malignancies when compared with controls. This indicates a particular vulnerability to cancer in people with mesothelioma. Not rarely, very old persons heavily exposed to asbestos remain free from asbestos-related cancer, a fact indicating an absolute resistance to the oncogenic effects of asbestos. A relative resistance may be recognized in people severely exposed to asbestos who develop mesothelioma only after 60 years or more since the onset of the exposure. The long survivals, rarely observed among mesothelioma patients, have been attributed to a high efficiency of immune mechanisms. Mesotheliomas have been reported among people with severe immune impairment, such as acquired immunodeficiency syndrome patients or organ transplant recipients. The natural history of mesothelioma shows that a resistance to the oncogenic effects of asbestos does exist. Probably, such a resistance is due to the efficient immune mechanisms. To strengthen the defence mechanisms may represent a way for preventing mesothelioma among people exposed to asbestos.

## INTRODUCTION

Asbestos,[1] erionite[2] and radiotherapy[3] are well known causes of malignant mesothelioma. Erionite may account for mesotheliomas occurring in some districts of central Turkey or developing among people migrant from this area. Previous radiotherapy may explain a small proportion of cases.[4] Some researchers have emphasized the possible role of non-asbestos causes.[5] However, at present, asbestos is considered by far the principal cause of mesothelioma, with 90-100% of the cases in some series being asbestos related.[4,6] Regarding the role of the different asbestos varieties, in particular chrysotile, in the induction of mesothelioma, the issue remains controversial.[1]

Mesothelioma incidence showed a dramatic increase in many industrialized countries during the last decades.[1] The highest annual crude incidence rates (30 cases per million and over) are reported from Australia, Belgium and the UK.[1] Rates comprised of between 11 and 23 cases per million are observed in large parts of Europe and in the US.[1,8] The geographical areas with the highest incidence/mortality rates correspond exactly to the sites of high shipbuilding activity and asbestos-cement production.[1] At a national level, a direct relationship has been observed between mesothelioma mortality and asbestos consumption occurrence during the previous decades.[7]

The mesothelioma epidemic has become a major health problem in various countries. In addition, millions of people heavily exposed to asbestos in the past are at a high risk of developing mesothelioma in the coming years. The objective of an early diagnosis is rarely reached in mesothelioma. Treatment is generally not efficacious.

In this context, it would be relevant to develop a better understanding of the mesothelioma genesis. While the importance of asbestos is undoubtful, the role of the host factors remains poorly recognized.[1] In the present review, some data on the role of individual susceptibility and resistance in the genesis of asbestos-related mesothelioma are discussed.

## SUSCEPTIBILITY

The existence of an individual susceptibility to the oncogenic effects of asbestos on the serosas is suggested by the fact that more mesothelioma cases have been repeatedly observed in the same family.[9–12] Blood-related subjects are mostly affected, a fact possibly indicating a genetically based vulnerability. Generally, patients with familial mesotheliomas have histories of exposure to asbestos, although cases without documented exposure have been reported.[12] The patrimony of observations collected in this field is not simple to evaluate and interpret. Generally, the reports of familial mesotheliomas are anecdotal, without any reference to a denominator. However, two studies conducted in Italy contain a reference group.[9–10] In the Trieste-Monfalcone area, a survey of 610 pleural mesotheliomas diagnosed since early 1970s revealed 40 familial cases.[9] Thirty-seven of these belonged to the original series of 610 cases. If only blood-related subjects were considered, and by excluding the families in which not all the members where comprised in the original series, the proportion of familial cases was about 4%. In a study comprised of three mesothelioma registries in Italy, 22 familial cases were identified among 1954 mesotheliomas,[10] with a percentage substantially lower than in the Trieste-Monfalcone area. The study of Ascoli *et al*.[10] included two registries of northern Italy (Brescia province and Veneto region) and one registry of southern Italy (Apulia region). This pooling could represent a confounding factor given the strong heterogeneity of the Italian populations.

In general, familial mesotheliomas do not differ in their natural history from the sporadic ones. This fact speaks against the relevance of genetic factors. Nevertheless, a 4% proportion of familial cases for a tumor that remains rare even among severely exposed populations should deserve further attention. Moreover, the data hitherto collected on familial mesothelioma are probably underestimated. In particular, the detection of the tumor in two generations may require very long observation periods.

In a recent study, the probability that the published familial clusters of mesothelioma could have randomly occurred in families exposed to asbestos was evaluated with the Family History Score Z_i_ (FHS_i_).[13] This analysis suggested that clustering may be explained with the additional contribution of other familial factors.

The association between mesothelioma and other malignancies in the same patient may represent another way of exploring susceptibility. The prevalence of such an occurrence has been evaluated variously. In a series of 169 necropsies reviewed in Monfalcone, Italy, pleural mesothelioma was associated with other, previous or synchronous malignancies in 32 cases (18.9%).[14]

In a recent study,[15] mesothelioma patients showed a greater risk of additional cancers when compared with other asbestos-exposed groups. These findings suggest that people with mesothelioma have an increased susceptibility to cancer.

Various data support the idea that the individual differences in susceptibility to mesothelioma might, at least partly, depend on differences in the metabolic genes involved in the detoxification.[16] An association has also been observed between polymorphism in DNA repair genes and asbestos-associated malignant mesothelioma.[17]

## RESISTANCE

Only small percentages of people severely exposed to asbestos develop mesothelioma.[18] This may partly to be due to the fact that mesothelioma development generally requires long latent periods.[19] In a series of 136 mesotheliomas of the pleura, investigated in the Trieste-Monfalcone area, the latency periods, defined as time intervals elapsing between first exposure to asbestos and diagnosis of mesothelioma, ranged from 25 to 71 years, with a mean of 48.8 years.[19] Many subjects exposed to asbestos die due to asbestos-related disease or other causes before the above long periods have elapsed. However, even old and very old people, with histories of ancient asbestos exposure and with large pleural plaques, may remain free from mesothelioma. A study conducted on a series of 3,600 necropsies shows that this is not an exceptional event.[20]

The formation of pleural plaques represents a nearly mandatory event among asbestos-exposed people.[20] This means that asbestos fibers constantly reach the pleura. The pleura constantly undergoes to an endless sequence of inflammation/repair processes. On the contrary, mesothelioma is the exception rather than the rule. The oncogenic properties of asbestos fibers may generally be neutralized in the serosas.

Besides this absolute resistance, a relative resistance to the oncogenic effects of asbestos on serosas may be recognized among those mesothelioma patients with very long latent periods. In such people, mesothelioma develops only 60-70 years after their first contact with asbestos.[19]

Some form of resistance may be recognized among the very rare mesothelioma cases with long survivals or recovery. Pilling *et al*.[21] recently reported one of these rare cases. The patient was asymptomatic 12 years after the initial presentation of a pleural mesothelioma. Obviously, the key question is what is the basis of the resistance. In their report, Pilling *et al*.[21] emphasize the fact that in their case a prominent inflammatory cell infiltrate was visible in the tumor at initial presentation. In the chest wall metastases, excised 5 years later, there was again a moderate inflammatory infiltration. The authors suggest that this represented a host response to the tumor and that such a response might be responsible for the favorable course of the disease.

In this connection, it should be remembered that a favorable course has been reported in lung carcinomas with a prominent lymphoid infiltration of the stroma.[22] In a recent study on nonsmall-cell lung cancer, a high number of CD8+ lymphocytes in the stroma of the tumor appeared as a favorable independent prognostic factor.[23]

The possible relevance of immune impairment in the genesis of the mesothelioma is also suggested by the observation of mesotheliomas in particular populations such as transplant recipients[24–25] and human immunodeficiency virus/acquired immunodeficiency syndrome patients.[26–27] A recent metaanalysis suggests that when a given tumor shows an increased incidence in such populations, immune deficiency probably plays a major role.[28] Mesothelioma does not appear in the studies analyzed by Grulich *et al*.[28] However, the development of such an uncommon tumor among some immunosuppressed patients remains noteworthy.

The role of immune surveillance suggested by the above facts could also be related to the mentioned coexistence of mesothelioma with other malignancies.

Interestingly, a study conducted in Wittenoom, Australia, an area severely polluted by asbestos in the past, found that the risk of developing mesotheliomas, due to environmental exposure, was lower among young subjects (less than 15 years) in comparison with older persons.[29] The authors of the research believe that this fact might be attributed to the existence of more efficient defence mechanisms among young people.

Regarding the role of immune mechanisms in the genesis of mesothelioma, a major point is that asbestos itself may affect T cells and NK cells.[30] Immune impairment caused by asbestos probably represents a relevant mechanism in the induction of asbestos-related mesothelioma as well as in the induction of asbestos-related lung carcinoma.

## CONCLUSIONS

Available evidence seems to indicate that the immune system is the principal basis for the susceptibility-resistance in asbestos-related mesothelioma. The identification of ways suitable for strengthening defence immune mechanisms in asbestos-exposed subjects should represent a priority goal for the research.
